# Supplementary material for: Prolonged contact with dendritic cells turns lymph node‐resident NK cells into anti‐tumor effectors
Source: EMBO Mol Med. 2016 Jul 12;8(9):1039–51. doi: 10.15252/emmm.201506164 (PMC5009809; doi:10.15252/emmm.201506164)
Supplement: Supplementary file 1 — Appendix [file EMMM-8-1039-s001.pdf]

Prolonged contact with dendritic cells turns lymph node-resident NK cells into anti-tumor effectors

Francesca Mingozzi[1], Roberto Spreafico[1], Tatiana Gorletta[1], Clara Cigni[1], Marco Di Gioia[2], Michele Caccia[3], Laura Sironi[3], Maddalena Collini[3], Matias Soncini[4], Michela Rusconi[1], Ulrich H. von Andrian[5], Giuseppe Chirico[3], Ivan Zanoni[1,2,4,6,7] and Francesca Granucci[1,4,6,7]

[1] *Department of Biotechnology and Bioscience, University of Milano-Bicocca, Piazza della Scienza 2, 20126 Milan – Italy*
[2] *Harvard Medical School and Division of Gastroenterology, Boston Children's Hospital, Boston, MA 02115, USA*
[3] *Department of Physics, University of Milano-Bicocca, Piazza della Scienza 3, 20126 Milan – Italy*
[4] *Humanitas Clinical and Research Center, via Manzoni 56, 20089 Rozzano (MI) – Italy*
[5]*Department of Microbiology and Immunobiology, Harvard Medical School, Boston, MA 02115, USA; The Ragon Institute of MGH, MIT, and Harvard, Cambridge, MA 02139, USA*

[5]equal contribution

APPENDIX TABLE OF CONTENTS

Appendix Figure S1	page 3
Validation of the ex vivo experimental conditions	pages 3,4
Appendix Figure S2	page 4
Appendix Figure S3	page 4
Analysis of DC and NK cell interactions	pages 5-15
Movie acquisition and tracking algorithm	page 5
Computation of dynamic parameters	pages 5-9
**NK-DC distance**	page 6
Equation 1	page 6
Equation 2	page 6
**Instantaneous velocity**	pages 6-8
Equation 3	page 7
Equation 4	page 7
Appendix Figure S4	page 8
**Confinement ratio**	pages 8,9
Equation 5	page 8
Equation 6	page 8
Equation 7	page 8
Appendix Figure S5	page 9
Equation 8	page 9
Operative definition of NK-DC interactions	pages 9-16
Appendix Figure S6	page 10
Appendix Figure S7	page 12
Appendix Figure S8	page 13
Appendix Figure S9	page 13
Appendix Figure S10	page 15
Appendix Figure S11	page 16

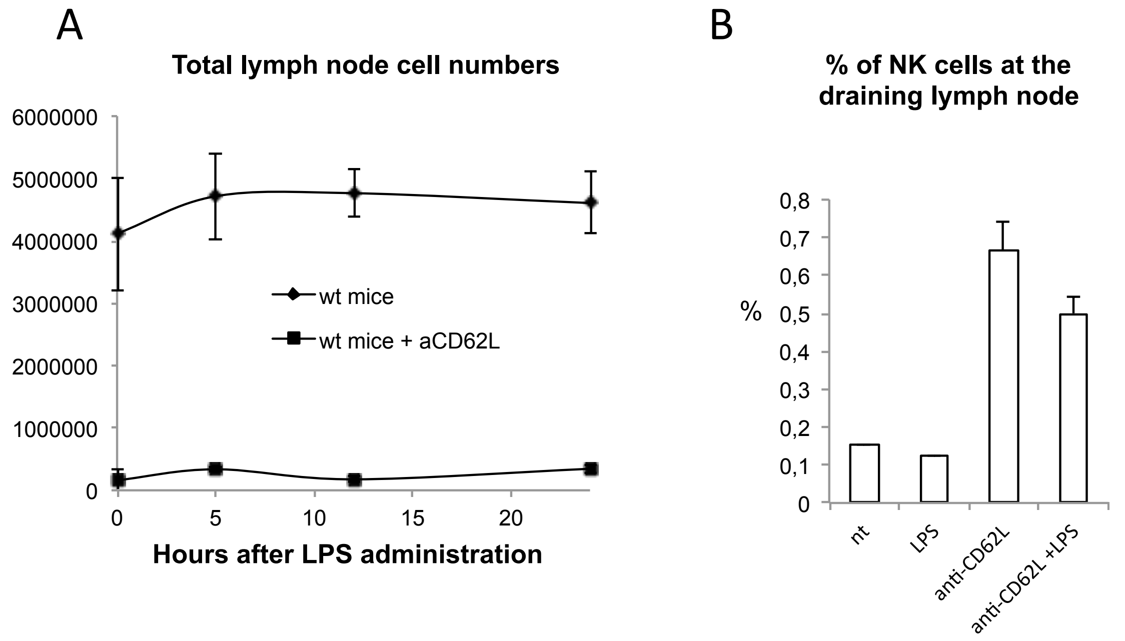

Appendix Figure S1. (A) Absolute numbers of cells in the draining lymph node before and at the indicated hours after LPS treatment in mice treated or not with the anti-CD62L antibody. (B) Percent of NK cells at the draining lymph node in mice treated or not with the anti-CD62L antibody to inhibit NK cell ingress in the lymph node from blood.

Validation of the ex-vivo experimental conditions.

For in vivo experiments, popliteal lymph nodes were surgically exposed and observed under the TPE microscope. Mice were kept anesthetized at 37°C during the measurements. For ex vivo experiments the explanted lymph node was kept in a chamber at 37°C and under a continuous flux of complete medium saturated with 95% O_2 – 5% CO_2 mixture.

The distribution of NK cells was then analyzed in the two experimental conditions. In both cases a preferential distribution of NK cells at the border of the T cell area was observed (Appendix Figure S2A, B).

Subsequently, movies of 3D volumes of lymph nodes in ex-vivo conditions for 30 - 90 min with a volume sampling time of 10 - 30 s were acquired. The movies were analyzed in order to segment NK and T cells and to reconstruct their motion. We focused the analysis of the cell traces (Appendix Figure S3a) on the evaluation of the 3D velocity that resulted $\langle v \rangle_{NKcells}^{ex-vivo}$ = 6.4 ± 2 μm/min (n=200 cells) for NK cells and that

resulted $\langle v \rangle_{Tcells}^{in-vivo}$ = 10 ± 2 μm/min (198 cells) for T cells (Appendix Figure S3b). These results were consistent with the data available in the literature for endogenous NK cells.

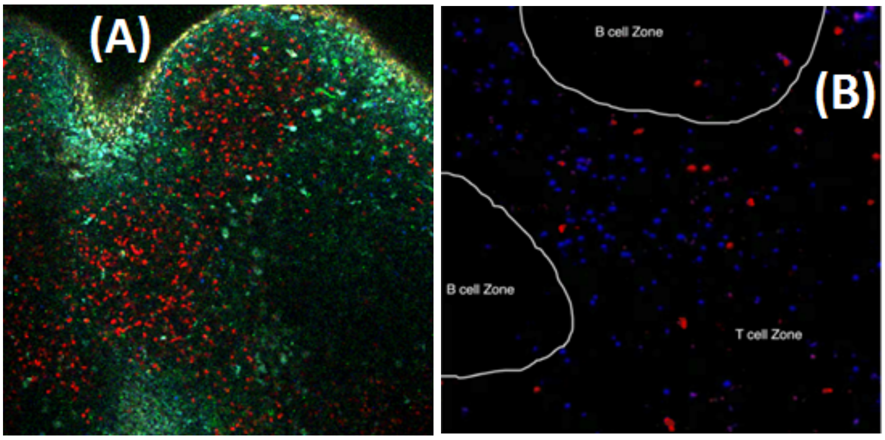

Appendix Figure S2. Distribution of NK cells in ex vivo and in vivo experiments. Left panel (panel A), ex vivo image of a brachial lymph node 72 h after B cell (red) and NK cell (blue) injection. Green cells are DCs. Right panel (panel B), in vivo image of a popliteal lymph node 72 hours after T cell (blue) and NK cell (red) injection. Note that in both cases NK cells distribute at the border of the T cell area. Image linear size = 750 μm.

(a)

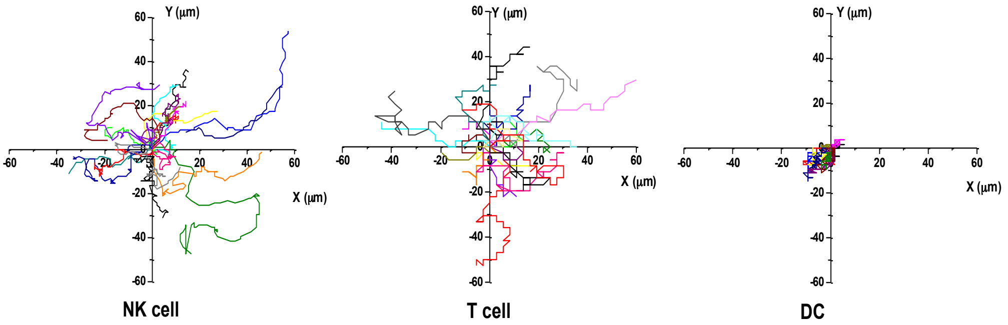

(b)

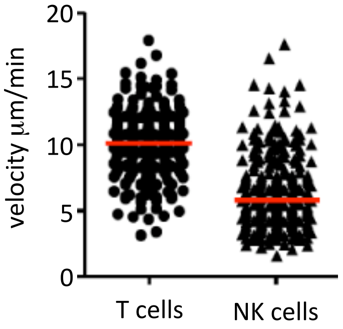

Appendix Figure S3. (a) *NK, T and DC trajectories in explanted lymph nodes tracked by Volocity software on the 4D volumes collected in lymph nodes on the TPE microscope*. (b) *Distribution of 3D velocities of NK cells in ex vivo conditions.*

Analysis of DC and NK cell interactions.

Movie acquisition and tracking algorithm.

The acquisitions contained a variable number (up to 500) of 3D volumes in which each stack was a 2D 256×256 pixels image. The z (optical axis) direction was sampled with 7-10 frames at a distance of 5 µm. The cell positions were interpolated in the z direction by weighting the intensity on each adjacent plane. The x-y field of view varied between 460×460 $µm^2$ and 700×700 $µm^2$.

The volume acquisition time (the minimum time between two xy frames at the same z height) was 10 - 30 s, depending on whether a Kalman filter (up to 5 frames) was applied during the acquisition. The cell motion was followed for 30-90 min under the TPE microscope.

Cells were segmented and tracked by means of the program Volocity (Perkin-Elmer). Both NK cells and DCs were tracked in a 3D volume representing a large fraction (up to 700×700×50 $µm^3$) of the lymph node. The frames in which a selected cell could not be segmented were disregarded during the analysis.

To perform cell tracking, we selected ROIs in the lymph node in which the density of cells (NK cells and DCs) allowed the discrimination of individual cells over time. The selection of the pool of NK cells that may interact with a selected DC was made according to a proximity consideration. Though DCs displayed much less motility than NK cells, we found necessary to keep track of their position over time in order to evaluate correctly the dynamic parameters (see below) that were used to estimate the duration of interactions.

Computation of dynamic parameters.

To characterize cell trajectories, we have computed and combined three parameters: the instantaneous velocity, the confinement ratio and the NK-DC distance. The rationale behind the choice of these parameters is that cell-cell

interaction is mediated by the emission of chemical signals, implying that NK cells lie in close proximity to a partner DC for a finite time. Hereafter, we briefly describe the definition and the meaning of these parameters.

NK-DC distance. The most obvious parameter that can be used to assess if an interaction is occurring between an NK cell and a DC is their mutual distance, *Dist(t)*. The threshold of NK-DC distance discriminating interacting and non-interacting events can be easily determined by looking at the size of NK cells (diameter 5-10 µm) and DCs (15-20 µm). For the latter, we should also consider the additional extension of dendrites (>10 µm [Bancherau 1998]). Taken together, we come to an estimate of D_{cutoff} = 25 µm as a cut-off value of the NK cell-DC distance. If the distance between a NK cell-DC couple lies below D_{cutoff} for the frames immediately preceding and following the i-th frame, then the two cells may be interacting in the i-th frame:

$$Dist(t_{i-1}) < D_{cutoff} ; Dist(t_i) < D_{cutoff} ; Dist(t_{i+1}) < D_{cutoff} \quad (1)$$

For visualization only, NK cell-DC distances can be digitized as follows:

$$\begin{cases} Dist_D(t_i) = -0.15 & \text{if } Dist(t_i) < D_{cutoff} \\ Dist_D(t_i) = +0.15 & \text{if } Dist(t_i) \geq D_{cutoff} \end{cases} \quad (2)$$

Instantaneous velocity, *v(t).* This parameter gives an estimate of the stepwise change in position of the cell (mainly NK cells) between consecutive frames. We expect that interacting cells display a reduced value of v(t) when compared to non-interacting cells. However, it must be considered that NK cells may constantly scan the DC surface, searching for receptors, even during productive interactions. As such, NK cells may still move around DCs at a significant speed, despite exploring a small space around the partner DC. This is pictured in Appendix Figure S4. As a result, the instantaneous speed may not be markedly decreased during the interaction. Consequently, while very fast or very slow events could be interpreted as non-interacting and interacting, respectively, with reasonable confidence, a "gray

area" of medium-slow events would comprise both true and false positive interacting events. The instantaneous speed, taken alone, would not be able to discriminate between the two, but it would still be helpful in filtering out a number of truly non-interacting events. Events classified as non-interacting using instantaneous speeds may have shown a more blurred classification based on other parameters. Reciprocally, other parameters may contribute to dissect true and false interactions in events all classified as positive using instantaneous speed alone. Therefore, there is value in setting a threshold of instantaneous speed above which events are surely non-interacting (true negatives), as long as this information will be consolidated with additional parameters to define interacting (positive) events with high confidence.

A conservative value of the threshold, v_{Thr} =60% of the NK cells mean velocity, was selected by averaging the instantaneous speed of all NK cells over all frames considered in a field of view (or movie). Therefore, as effect of the threshold filter, the instantaneous digitalized velocity becomes:

$$\begin{cases} v(t_i) = 0 \quad \text{if} \qquad v(t_i) < v_{thr} \\ else \\ v(t_i) = v(t_i) \end{cases} \tag{3}$$

For visualization only, we normalized the instantaneous speed to its maximum value, $v_{Norm}(t_i) = v_{Norm}(t_i) / v_{\max}$.

In Appendix Figure S5 we illustrate the threshold filter on the instantaneous speed trace of a representative NK cell. We labeled the i-th frame as a putative NK-DC interaction if the instantaneous speed lies below the threshold value in the immediately preceding and following frames:

$$v(t_{i-1}) < v_{thr} \quad \cap \quad v(t_i) < v_{thr} \quad \cap \quad v(t_{i+1}) < v_{thr} \tag{4}$$

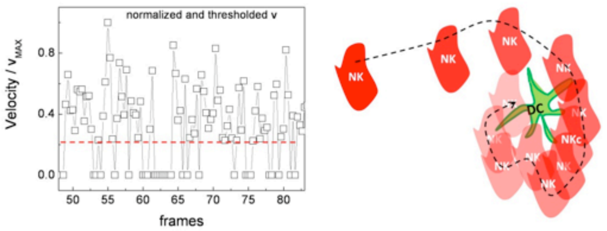

Appendix Figure S4. Schematic of the motion of an NK cell moving towards and interacting with a DC. The fading red color indicates successive frames in the movie. The plot reports a typical evolution of the instantaneous speed (normalized to the maximum value) for an NK cell as a function of the frames acquired in the movie. The red dashed horizontal line indicates the threshold value (for the choice of this value please refer to the text). All the values that lie below the threshold are set to zero in the plot.

Confinement ratio. In the schematic reported in Appendix Figure S5, it may be seen that interacting NK cells, in addition to slightly reducing their instantaneous speeds, lie in a confined volume around the DC for a finite time. To model this, we borrowed the concept of end-to-end distance of a polymer from materials science, and applied it to cell traces.

The cell position is defined by a 3D time vector $\{\mathbf{r}(t_i)\}_{i=1,M} = \{x(t_i), y(t_i), z(t_i)\}_{i=1,M}$ where t_i is the time corresponding to the i-th frame and M is the number of frames over which the NK cell was tracked. We define the instantaneous displacement (analogous to the end-to-end distance of a polymer of i monomers) as:

$$D(t_i) = |\mathbf{D}(t_i)| = |\mathbf{r}(t_i) - \mathbf{r}(t_1)| \quad (5)$$

The instantaneous contour length of the track is defined as:

$$C(t_i) = \sqrt{\sum_{i=1,M-1} |\mathbf{r}(t_{i+1}) - \mathbf{r}(t_i)|^2} \quad (6)$$

The confinement ratio $d(t_i)$ is then defined as the ratio of the displacement and the contour length:

$$d(t_i) = \frac{D(t_i)}{C(t_i)} \quad (7)$$

An example of d(t) for typical interacting and non-interacting events is given in Appendix Figure S5.

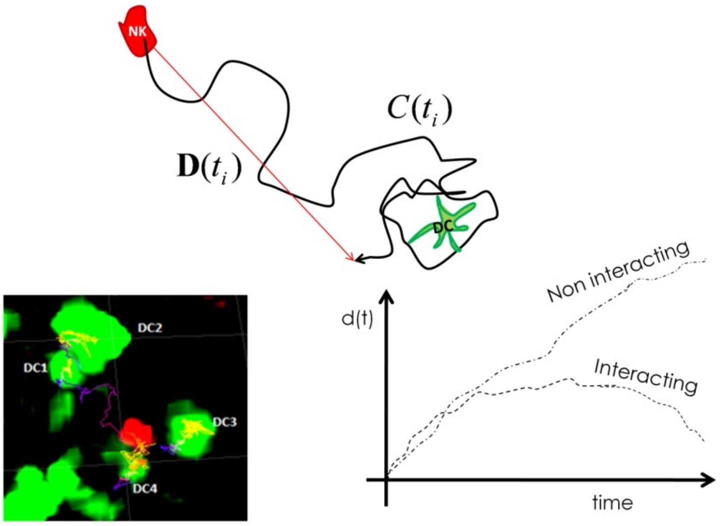

Appendix Figure S5. Graphical schematic of the definition of the confinement ratio, d(t), as a function of the time along the movie. The definitions of $\mathbf{D}(t)$ *and* ***C(t)*** *are given in* ***Eqs. 3, 4****. The image (linear size = 55 μm) reports a snapshot of a movie depicting one NK cell (in red) and four DCs (DC1 to DC4) in green color. The trajectory of the NK cell is traced in the image in blue color. The plot reports the typical trend of the confinement ratio for a NK cell interacting (dashed) or not (dot-dashed) with a DC.*

When cells productively interact, the NK cell should move in close proximity of the partner DC for a finite time. This behavior would imply a decrease of the confinement ratio, as depicted in the plot of Appendix Figure S5. We labeled the i-th frame as a putative NK-DC interaction if the confinement ratio decreases in successive frames, so to satisfy:

$$d(t_{i+1}) < d(t_i) < d(t_{i-1}) \qquad (8)$$

Operative definition of NK-DC interactions.

After LPS administration, we observed a marked change in the behavior of the NK cells that assumed longer and more stable contacts (Δt>15 min) with DCs with respect to the steady state condition. We provide hereafter an example of the multi-parametric analysis done for the interactions of DC and NK cells.

Appendix Figure S6 (panel A) shows an example of a long-lasting contact between an NK cell and a DC. During its motion the NK cell seems to recognize an activated

DC probably due to chemical attraction (Appendix Figure S6A, panels A and B), subsequently it contacts the DC (Appendix Figure S6A, panel C) and forms a temporarily stable contact with it (Appendix Figure S6A, panels D, E and F). Even if, once encountered the DC, the contact is dynamic with the NK rocking and rolling around the DC, its motion becomes confined and, as consequence of the long lasting of the contact ($\Delta t > 15$ min), the dynamic parameters change significantly during the interaction. The NK slows down its mean velocity changes from 5.5 to 4 µm/min and its instantaneous velocity has more zeros than in the first part of the track (Appendix Figure S6B). The distance covered by the NK cell from 40 min to 61 min is 17 µm compared to the 36µm distance made overall during the 30-40 min time interval. Also the confinement ratio covered decreases abruptly (0.3 vs 0.07). These changes in the cellular motion, as depicted in Appendix Figure S6, can all be ascribed to a shift from a free diffusion to a confined motion: they, taken together with an AND condition, define an interaction.

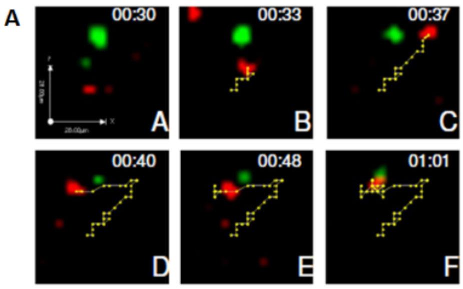

B

Time [min]	N° zeros	<v> [µm/min]	Disp. [µm]	CR
30-40	2	5.5	36	0.3
40-61	13	4	17	0.07

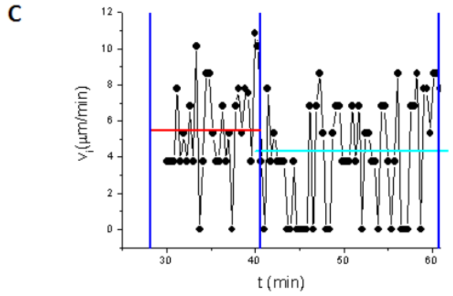

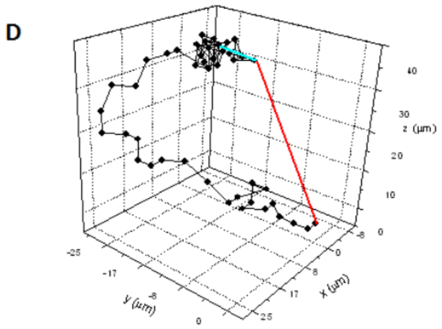

Appendix Figure S6. Interaction between an NK cell (red) and a DC (green) after LPS administration. (A) In the panels A, B and C the NK cell moves according to a directional random motion while in the panels D and E it moves toward the DC. Panel F shows the stable contact DC-NK: it is noteworthy the time lapse between D (starting of the interaction) and F (established interaction) is 21 minutes. We consider stable this type of contact. (B)Table of the physical parameters selected to define the interaction. Disp=Displacement, CR = Confinement ratio. (C) Instantaneous velocity of the trajectory; the mean velocity before (red line) and during (cyan line) the interaction are reported. The number of zeros reported in panel B is calculated from this graph. (D) 3D rendering of the trajectory followed by the NK

cell. The red and the cyan line represent the displacement before and during the interaction, respectively.

We have devised an algorithm that works simultaneously on the cell speed, the trajectory confinement ratio and the NK-DC distance. This allows us to analyze the trajectories searching for possible contacts and it can be summarized as follows:

1. The speed of the NK cell must be at most 60% of the average speed,

$$v(t_i) < 0.6\langle v \rangle$$

2. the confinement ratio CR must decrease or stay constant
3. $Dist(t_{i-1}) < D_{cutoff}; Dist(t_i) < D_{cutoff}; Dist(t_{i+1}) < D_{cutoff}$. In this case a flag, called $T_{on}T_{off}$, is raised from the basal level $T_{on}T_{off}$ = -1, in absence of interactions to $T_{on}T_{off}$ = + 1.

In order to identify a contact, the parameters have to obey the constraints reported above simultaneously. In the case that even a single parameter does not fulfill its corresponding requirement, the NK cell is not considered as interacting with a DC. We summarize in Appendix Figure S7 the use of the three kinematic parameters.

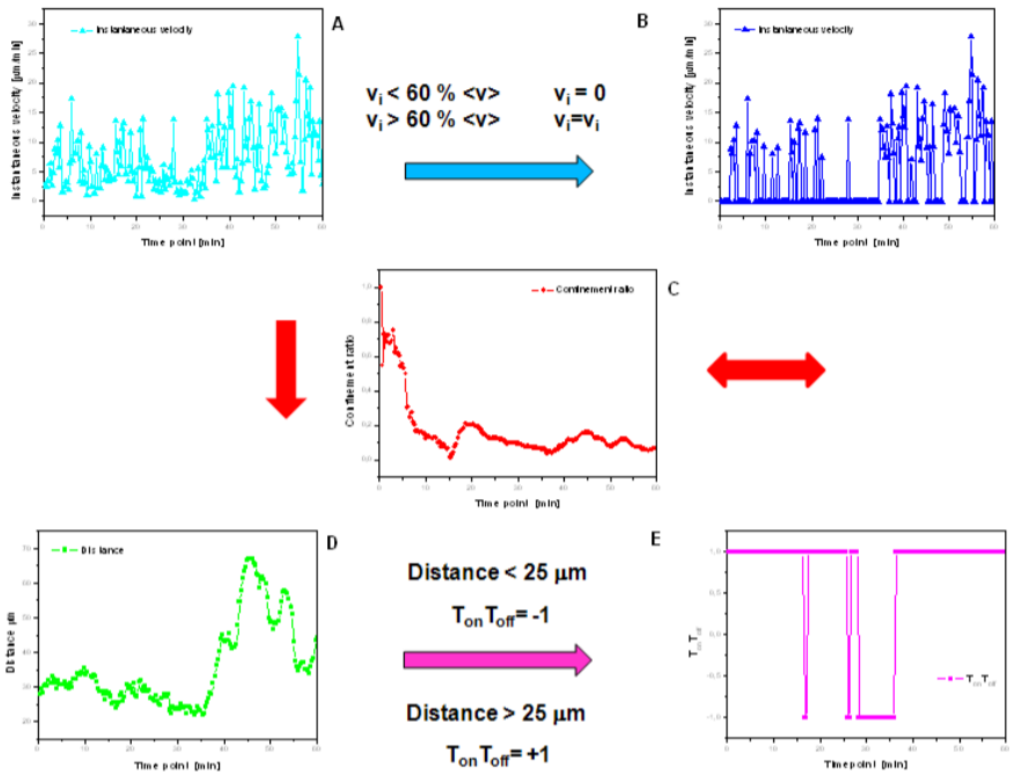

Appendix Figure S7. Parameters used to evaluate DC-NK interactions. Panels (A) and (B) report the plot of the NK cell instantaneous velocity before and after the threshold evaluation. Panel (C) shows the value of the NK cell confinement ratio as a function of time. Panels (D) and (E) display the NK-DC distance value as a function of time and the TonToff parameter, obtained as described in the text.

Examples of interaction assignment and fake assignments.

We show here that our algorithm is more severe that others employed in the literature.

In Appendix Figure S8, the panel A shows the screenshot of the acquired movie where a NK cell is near a DC. In the graph of panel B the four parameters, instantaneous velocity (blue), confinement ratio (red), $T_{on}T_{off}$ (magenta) and distance (green) are normalized in order to visualize them in the same graph. Even if the distance NK-DC1 is < 25 µm at the time points 55-58, the velocity is not < $0.6 \cdot \langle v \rangle$ and the confinement ratio increases.

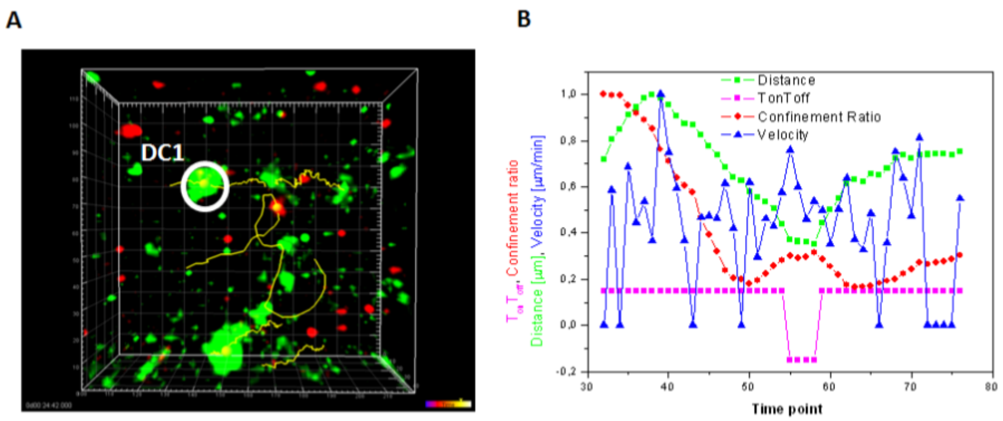

Appendix Figure S8. Panel A reports the screenshot of the acquired movie of an NK cell and a DC. The panel (B) shows the graph in which the four parameters (distance, instantaneous velocity, confinement ratio and $T_{on}T_{off}$) of the NK-DC1 couple are simultaneously reported. In order to visualize them the instantaneous velocity and the distance are normalized and the $T_{on}T_{off}$ parameter is rescaled. In the case of DC1, even if the distance NK-DC1 is <25 µm at the time points 55-58, the velocity is not < 0.6 < v > and the confinement ratio increases. Each time point corresponds to 24.7 s. The image dimension is 115x115x45 µm^3.

A different couple of cells is reported in Appendix Figure S9. Even if the distance NK-DC1 and the velocity respect the threshold, the confinement ratio increases at the time point 170-171. Otherwise, at the point 185-186, the distance respect the constraint and the confinement ratio decreases, but the velocity is > $0.6 \cdot \langle v \rangle$.

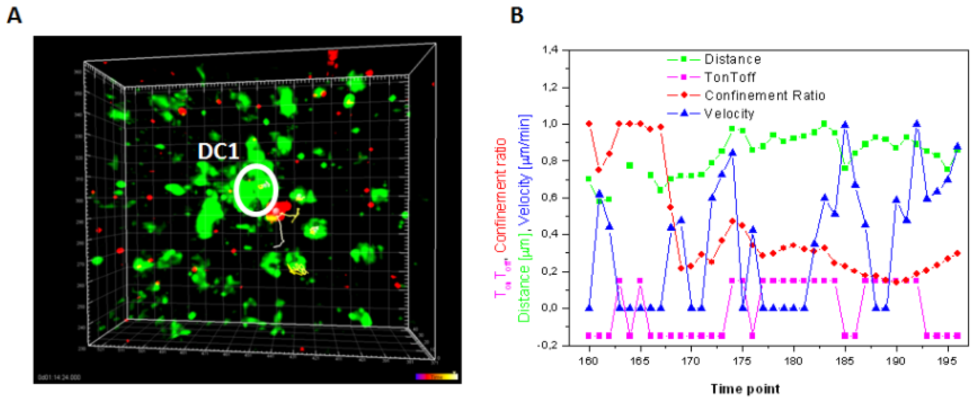

Appendix Figure S9. Panel A: screenshot of the movie of an NK cell and a DC. Panel (B) shows the plot of the four parameters (distance, instantaneous velocity, confinement ratio and $T_{on}T_{off}$) for the NK-DC1 couple reported at the same time.. In order to visualize them simultaneously, the instantaneous velocity and the distance are normalized and the $T_{on}T_{off}$ is rescaled. Even if the distance NK-DC1 and the velocity respect the threshold (i.e. distance NK < 25 µm) and $v(t_i) < 0.6 \cdot \langle v \rangle$), the confinement ratio increases at the time point 170-171. Otherwise, at the time points 185-186, the distance respect the constraint and the confinement ratio decreases, but the velocity does not $< 0.6 \cdot \langle v \rangle$. In both cases we infer that

the NK cell does not interact with the DC1. Each time point corresponds to 24.8 s. The image dimension is 155x135x45 μm^3.

Finally in the panel A of Appendix Figure S10 we have the screenshot of an NK cells near two DCs. Panel B shows that, at the time points 102-103, v(t_i) < $0.6 \cdot \langle v \rangle$ and the confinement ratio decreases, but the distance is > 25 μm. As consequence the NK is not interacting with DC1. The conclusion is the same for the NK-DC2 couple (panel C): at the time points 93-94 and 110-113, the instantaneous velocity is < $0.6 \cdot \langle v \rangle$ and the confinement ratio decreases, while the distance is above the threshold.

In all these exemplary cases at least one of the selected parameters does not fulfill the constraints of the algorithm. In Appendix Figure S8 the NK cell does not slow down and it follows a quite straight trajectory. In Appendix Figure S9, the confinement ratio or the instantaneous velocity parameters exclude the cells under investigation from the group of the interacting ones, while in Appendix Figure S10 the distance constraint is not respected. No one of the cells in Appendix Figure S8-S10 can be assigned to the group of interacting leukocytes.

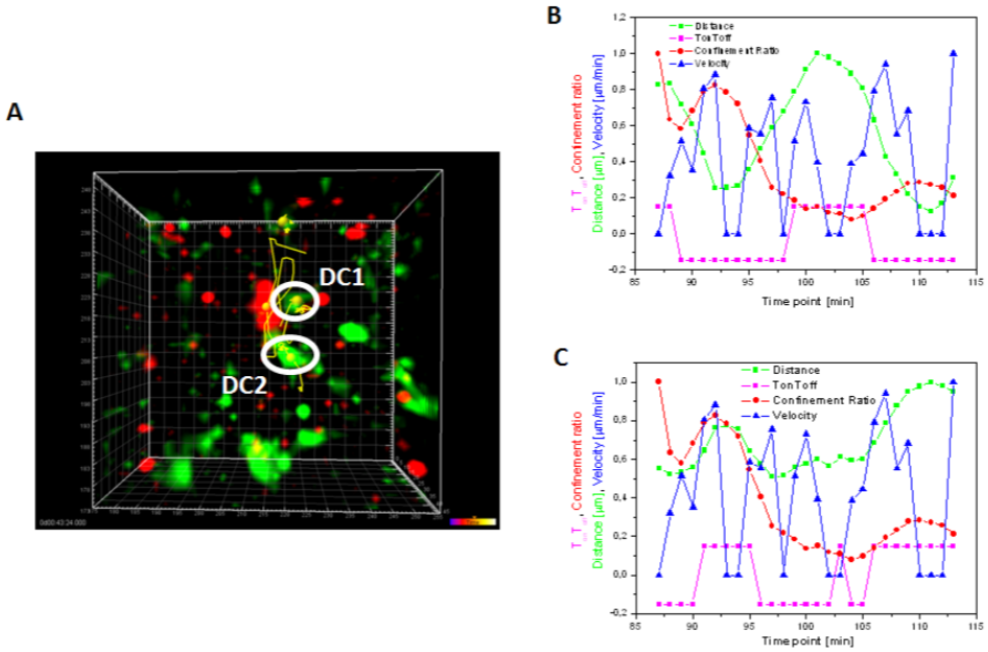

Appendix Figure S10. (A) Screenshot of the acquired movie of an NK cell near two DCs. Panel B shows the graph in which the four parameters (distance, instantaneous velocity, confinement ratio and $T_{on}T_{off}$) of the NK-DC1 couple are reported together. In order to visualize them simultaneously, the instantaneous velocity and the distance are normalized and the $T_{on}T_{off}$ is rescaled. At the time points 102-103 $v(t_i) < 0.6 \cdot \langle v \rangle$ and the confinement ratio decreases, but the distance is > 25 µm; as consequence the NK cell is not considered interacting with DC1. (C) Also for the NK-DC2 couple, at the time points 93-94 and 110-113, the instantaneous velocity is $< 0.6 \cdot \langle v \rangle$ and the confinement ratio decreases, while the distance is above the threshold. We can then infer that the NK cell is not interacting with the DC. Each time point corresponds to 24.8 s. The image dimension is 80x80x45 µm^3.

Only when the four parameters are verified simultaneously, the NK cell is assumed to interact with the DCs and the touch-time is quantified.

Appendix Figure S11 shows an example where the constraints on $v(t_i)$, the confinement ratio and the distance are verified at the same time: the cells are assigned to the interacting group and the Δt during which the constraints are all fulfilled is defined as the duration of the interaction (green box in Appendix Figure S11).

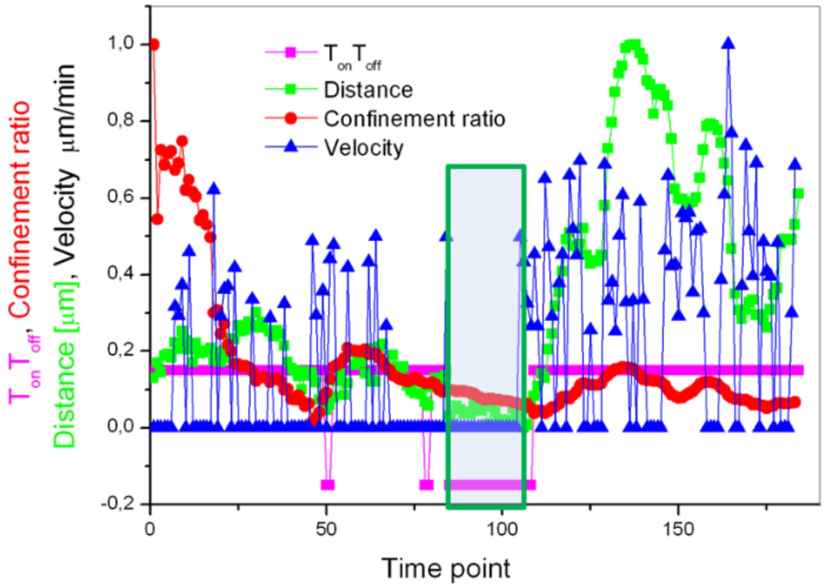

Appendix Figure S11. Plot showing the superposition of the parameters used to evaluate an NK-DC interaction: confinement ratio (red), threshold instantaneous velocity (blue), NK-DC distance (green) and $T_{on}T_{Off}$ parameter (magenta). The green box highlights the time lapse in which the interaction between NK cells and DCs occur because the parameters obey at the same time the constraints reported in the text.

Within the application of the algorithm described above, we have assumed that an identified DC-NK cell contact may fail for at most one frame (that is 10s - 30s) in at most one of the three dynamic parameters. Therefore after the whole trajectory was scan with the algorithm we have rescan the computed data sets of interaction durations to join those durations that where interleaved by a single frame in which a single parameter failed to satisfy the constrains. It must be also considered that the frame duration was comparatively low with respect to the duration of the interactions typically detected.
