# Supplementary material for: Prolonged contact with dendritic cells turns lymph node‐resident NK cells into anti‐tumor effectors
Source: EMBO Mol Med. 2016 Jul 12;8(9):1039–51. doi: 10.15252/emmm.201506164 (PMC5009809; doi:10.15252/emmm.201506164)
Supplement: Supplementary file 2 — Movie EV1 [file EMMM-8-1039-s002.zip › Video1/1.docx]

Video 1. DCs and NK cell dynamic behavior in resting conditions. CD11c.GFP mice were adoptively transferred with 5 x 10^6^ CMTPX-labeled NK cells. Intact brachial lymph nodes were explanted one day later and subjected to two-photon imaging. Time-lapse movies were processed to fully discriminate GFP from CMTPX signals. DCs are pseudocolored in green and NK cells in red. Total duration 36’. Grid square 7 x 7 μm.
