# Supplementary material for: Prolonged contact with dendritic cells turns lymph node‐resident NK cells into anti‐tumor effectors
Source: EMBO Mol Med. 2016 Jul 12;8(9):1039–51. doi: 10.15252/emmm.201506164 (PMC5009809; doi:10.15252/emmm.201506164)
Supplement: Supplementary file 3 — Movie EV2 [file EMMM-8-1039-s003.zip › Video2/Video2.docx]

Video 2. DCs and NK cell dynamic behavior in resting conditions. CD11c.GFP mice were adoptively transferred with 5 x 10^6^ CMTPX-labeled NK cells. Intact brachial lymph nodes were explanted one day later and subjected to two-photon imaging. Time-lapse movies were processed to fully discriminate GFP from CMTPX signals. DCs are pseudocolored in green and NK cells in red. Total duration 44’. Grid square 20 x 20 μm.
